# The traumatic consequences of Boko Haram slavery among the ethnic minorities of southern Borno, Borno State, Nigeria

**DOI:** 10.4102/phcfm.v14i1.3638

**Published:** 2022-12-15

**Authors:** Samaila Ziradzo, Robert T. Netangaheni

**Affiliations:** 1 Department of Sociology, College of Human Sciences, University of South Africa, Pretoria, South Africa; 2 Department of Health Studies, Faculty of Health, University of South Africa, Pretoria, South Africa

**Keywords:** Boko Haram, southern Borno, Chibok, ethnic minority, genocide, trauma, slavery, abduction

## Abstract

**Background:**

The investigation on the experiences of Kibaku ethnic minorities of southern Borno in the Federal Republic of Nigeria under the Boko Haram (BH) insurgency is inevitable, considering the dire humanitarian situation that has since prevailed. The mass massacre indicates the violation of human rights.

**Aim:**

To explore, describe and analyse the BH insurgency in north-eastern Nigeria and its traumatic effects which may be experienced at several interrelated levels.

**Setting:**

The research was conducted within the Kibaku ethnic communities of Chibok local government areas of Southern Senatorial District in Borno State of Nigeria.

**Methods:**

A predominantly exploratory and descriptive qualitative research design approach was adopted with a total of 80 participants.

**Results:**

The health consequences of BH-inspired victimisation include sexually transmitted diseases such as AIDS and chronic infections, unwanted pregnancy, miscarriage and other reproductive health problems. The psycho-emostional effects are both incalculable and unquantifiable, which is compounded by grief for the loss of victims through either abduction or death.

**Conclusion:**

The unjustifiable mass violence against the minorities may, from a historical viewpoint, be an orchestrated suffering of humans at the hands of other ‘humans’ in north-eastern Nigeria.

**Contribution:**

This article will add to the body of knowledge considering the magnitude (scale) and implications (scope) of the genocidal violence meted by a motley of sectarian malcontents propagating some illogical, unscientific, and historically ideologies bothering on a combination of racial bigotry, ethnicity and religious intolerance in Nigeria.

## Introduction

The Kibaku communities are ethnic minority groups living in the Southern Senatorial District of Borno State. They are adherents of Christianity, African traditional religion, and for a few, Islam. Following the upheaval in north-eastern Nigeria, an Islamic fundamentalist militant group, also known as Boko Haram (BH), declared an Islamic caliphate in north-eastern Nigeria.^1^ On 24 August 2014, BH fighters captured Gwoza, a town in southern Borno, declared it the headquarters of the new Islamic caliphate and made a public statement to purify their caliphate of non-Muslims and non-Sunni Muslim ‘apostates’.^2^ This strident message exacerbates within-Muslim tensions and worsens non-Muslim and Muslim relationships in the north-eastern region.^3^ Moreover, they committed numerous atrocities and violations against the civilian population, and due to their identity, the ethnic minority communities who were targets and the most severely affected communities within the region.^4^

In September 2014, BH attacked the ethnic minority ancestral homelands of Chibok; during the attacks, BH mutilated, murdered, abducted and enslaved hundreds of men and women, including children, displacing entire communities to Internally Displaced Persons (IDPs) and refugee camps in the process.^5^

One of BH’s most traumatic offences was the abduction and forceful conversion of 276 school girls at Chibok in February 2014. Consequently, the group captured and placed under its control, a large part of north-eastern Nigeria territories, including the southern Borno district. Due to the nature of their onslaught, one was known as the ‘Uziri Genocide’ at Madagali.^6^ Nevertheless, the minorities have suffered centuries of discrimination, marginalisation and persecutions through *jihadist* persecutions at various points in history.^7,8^

Reynolds (2001) alludes that the promotion of Islam through the Indirect Rule in Northern Nigeria strengthened the powers of the Muslim rulers as legitimate traditional rulers (junior partners), while politically marginalising the minority counterparts.^9^

These differences escalated and became more pronounced from the 1930s to the 1950s, with the advent of Christian evangelism by missionaries among the non-Muslim communities. When decolonisation gained its impetus in the 1950s, the general language of Christianity gradually served to weaponise the politico-religious mobilisation against Hausa-Fulani Muslim hegemony.^10,11^

Generally, survivors of terrorist attacks are known to have suffered post-traumatic stress disorder more frequently than survivors of motor accidents.^12^ Among previously captured women and girls, perceived social rejection in their community conciliates the relationship between traumatic enslavement events and depression symptoms.^13^ Some may include replays of carnage, flashback and nightmares, which may persist for months or even years.^14^

## Methods

The qualitative method, utilised as a naturalistic approach, seeks to unearth participants’ opinions, thoughts, experiences and feelings.^14^ They consider the study’s main purpose, which is to create an awareness that relevant victims have a right to live, to be heard, to freedom of belief and to justice and to encourage them to deal with the tragedy that befell them.

### Setting

The data were collected within the Kibaku communities of Chibok local government areas of Southern Senatorial District in Borno State, Nigeria. The local government area has an area of 1350 km^2^, with a population of 93 200.^15^ They speak ‘Kibaku’, one of the Biu Mandara languages (Central Chadic) spoken today from the island of Lake Chad to the valley of the River Benue.

### Sampling strategy

The sample was drawn from the BH survivors, 18 years of age and older from the Kibaku communities of Chibok local government area, hosting hundreds of IDPs in Southern Borno, the Borno State of Nigeria. The study’s sample size was made up of 80 participants. Of these, 46 participated in semi-structured interviews, while 34 were involved in seven focus group discussions (FGD).

### Inclusion criteria

Survivors within Kibaku communities that were 18 years and above.

### Exclusion criteria

Survivors who did not consent to the study.

### Data collection

The researchers collected data from the Kibaku communities of Chibok by using in-depth interviews and FGDs as primary instruments for collecting information.

All interviews and FGDs with encamped displaced persons and survivors were conducted with the permission of the host community’s leaders and government authorities. Access to self-settled survivors was further arranged with the host community leaders and humanitarian volunteers. High-profile survivors (clergy or senior government officials) who attracted media attention while in captivity, and still under restriction were accessed after consultation with religious authorities.

### Data analysis

Thematic data analysis was employed to collate all the information accrued from both the semi-structured interviews and the FGDs. Ultimately, the findings from both data sets were manually converged for the purpose of generating global themes of the study as a whole.

A specified coding was created for similar themes; they were arranged into research questions, and integrated into themes and sub-themes of the study, based on the order of evidence emanating from the themes and sub-themes of the research findings.

### Ethical considerations

This article is part of a PhD dissertation, ‘An Investigation of the Experiences of non-Muslim Communities of southern Borno Under the BH Insurgency in the Borno State of Nigeria’. It was approved by the Research Ethics Committee of the College of Human Science (CHS), University of South Africa, with reference number 2021-CHS-68965605.

## Results

### Theme 1: Violation of non-Muslim women and girls

The study findings showed that the atrocities committed against ethnic minority women and girls have led to expulsion, forced migration and exile from their ancestral lands in north-eastern Nigeria.

The women’s and girls’ lives in captivity have been the unreported victims’ experiences of the BH war for over a decade. The Islamic group tactically and deliberately targets minority women and girls for abduction, gender-based violence, and doing so, with impunity.

The following sub-theme was created from Theme 1.

#### Sub-theme 1: Targeting non-Muslims for abductions

The incident of 276 schoolgirls being abducted in Chibok in 2014, had sparked international indignation. Although some survivors are rebuilding their lives, more than a hundred are still in captivity.

One of the schoolgirl survivors in her narration revealed:

‘I do not know what hell fire is like, but a day in BH captivity may be worse than 100 days there for a non-Muslim captive [*at the time of abduction Muslims are separated and set free*]. Some Muslim women treated us worse than their husbands did to us. Those women were holding non-Muslims girls for their husbands to assault, rape and torture. [*It’s so traumatic that*] they shall not escape justice. We all wanted them punished too … mass abductions of school children in this country have been making international headlines lately. It has been very hard for me to watch the news; remembering our abduction at government school at Chibok, in April 2014. It makes me remember how I survived one of the most tormenting experiences a teenager could undergo.’ (Female, pt. 3, 24 years old)

‘We were about 40 Kibaku-Chibok women and girls kept as a [*sex*] slaves for 2 months, in a place called Shuwa in Adamawa …they showed us video of the girls they abducted earlier in government secondary school at Chibok and vowed that, it will soon be part of Islamic caliphate.’ (Female, pt. 32, 30 years old)

‘They killed them all in front of my eyes: my God-fearing daddy, uncle, my four cousins, some relatives from Bwaftari … they covered my cousins’ eyes and tied their hands and cloths to each other, took them near to a river in Lassa. I suddenly heard a gun-shot; subsequently, they said “mun kasha arnan” (we have killed the infidels).’ (Female, pt. 32, 30 years old)

‘I never wanted to recall what happens to me and my family, but I seek justice for the innocent violated non-Muslims girls. I seek only justice for my aged father, cousins and uncle, and finally for those that remain in captivity with the Islamic sect all over.’ (Female, pt. 32, 30 years old)

#### Sub-theme 2: Sexual slavery and torture of non-Muslims

‘Thousands of our women and girls were involuntarily forced into sexual slavery by BH terrorists. At a point we were over 50 Kibaku women and girls in a particular place in Sambisa … said survivor that was in captivity for 6 years.’ (Female, pt. 28, 38 years old)

‘We were kept in a very big upstairs house in an open place with lots of non-Muslim girls crying and traumatised … a selected group of girls were taken, to be presented as a “gift” to “Amir” at Gwoza … the “Amir” distributed us to some strange fighters with long hair. I openly rejected his proposal, and I was beaten to pulp, and tied up on a tree for 2 days without food. They dug a hole where the normally put women who resisted them to be stoned to death.’ (Female, pt. 07, 26 years old)

‘… you are the one talking to me you are the family I know, my family for now are unknown, whether alive or death, and my whole village is deserted there is no single Soul.’ (Female, pt. 07, 26 years)

‘They did to me what they did to many Kibaku girls. For 3 years, I was tortured, beaten with iron pipe and tied up on a tree, raped multiple times. The unforgettable, was the one aided by two Muslim fighters from my community. Those boys tied me up and held me down for a dirty fighter to rape me, while watching and laughing … I rejected conversion and forceful marriage, but I paid dearly with constant rape, beaten and left me unconscious many times. Before my escape I was tied on a fallen tree beaten and left to die, all these scars on my body, is what I went through … they were all tortured and raped despite conversion. The male factors were mostly Kanuri-Muslims and former Al’majiri’s. They brought a girl of 12 years to our place and molested her; they wanted her to marry a man of more than 50 years, but at the end, he was killed in one of their attacks. They beat her to a pulp, saying that she is possessed with evil spirits; she is the cause of his death. They dug a hole and bury her leaving only her head outside and left her. They said at evening time, she would be stone to death. Unfortunately, there was heavy rainfall. By midnight, one of the Muslims fighters from my community came and untied me and pull out the girl from the grave hole. He said this may lead to his death, but I must leave the place. That is how he save us, and we escaped.’ (Female, pt. 08, 27 years old)

‘I carried my two children with me, and my pregnancy was visible but one of the fighters chose me to be his wife. He told me, if I did not consent to his proposal, he would send me to Gwoza and marry another man who would take me to Cameroon. At the end he forced himself on me, while his wife was holding my hands down as he raped me. He said the child will be burn in the caliphate and will be raised as next generation of fighters. Before … his initial threats of keeping my child to become a terrorist, he was killed in one of the attacks. And one of his wives helped me by introducing me to her brother who is a commander … he married me and after sometimes she aided my escape from the forest.’ (Female, pt. 26, 30 years old)

#### Sub-theme 3: Forced conversion, marriage for non-Muslims

The non-Muslim women and girls abducted by the Islamist jihadist group BH are forced to convert to Islam, marry and endure physical and psychological abuse in captivity. The study showed that marriage was conducted in BH captivity without seeking the consent of the bride, usually obtained through emotional pressure or intense physical abuse.

A woman revealed,

‘Conversion to Islam was a pre-requisite for survival as a female captive. I told one of the fighters that I would not marry a killer of the innocent, he pulled out a gun and hit me with it on my forehead, [*the scar was very clear on her forehead*]. The fighters told me … are you better than the schoolgirls we abducted and married from Chibok? The Chibok girls have now understood the religion and can behead or cut off the throats of their parents, if, they ever see them. He immediately married me off to one of the foreign fighters from Mali.’ (Female, pt. 07, 26 years old)

Another participant disclosed,

‘A pregnant woman was forced to marry a fighter in a village near Gwoza (caliphate headquarters); she pleaded, as her pregnancy was clearly obvious. She then spit in the face of the fighters, which is usual for pregnant women but was punished by BH fighters. They pushed her out in the sun and marched on her head and her stomach. They kicked her so badly and was covered in blood (she had miscarried) as a result … after 2 days she was forced to marry a fighter against her will … and she died of internal bleeding. One of the foreign fighters was angry with the incident, he became overwhelmingly disgusted and decided to flee the group. He opposed the leader, that the act was un-Islamic.’ (Female, pt. 13, 26 years old)

Women and girls held captive by BH were forced to convert to Islam:

‘Boko Haram views Christians and other religions as infidels and considers non-Muslims unkempt unclean and unworthy of existing.’ (Females, pt. 07; 08; 13; 26; 28)

‘We were also asked by insurgents to forget about any of our past religion and non-Muslim relatives, that all are considered unclean before Allah … that, non-Muslims are not allowed to visit Islamic Holy land because they are untidy. We were forced to repeat the Kalmar sha’hada (Islamic creed), recite from the Quran, and collectively, we are told that we are converted to Islam … given the choice of getting married or serve as domestics slave to fighter’s wives. Many chose to serve as slave but that does not stop the insurgents from raping and torturing them. I was forced to marry a fighter before I escaped.’ (Female, pt. 26, 30 years old)

Another survivor revealed:

‘… [*T*]he BH forcefully took us, kept us as captives, and forcibly married us against our will. What pained me the most was seeing my own daughter also being sexually violated and married, and I could not do anything to help her out … consoling her was even hard for me. I did not know how to encourage her because I was undergoing same as well. I also went through such a horrible experience and we both have babies for BH fighters …By separating us from our men and forcibly marrying and impregnating women. The only way you can re-unite with your husband [*was by both converting to Islam and subsequent recruitment into the caliphate r ijale fighters*]. Boko Haram terrorists are preventing another generation of non-Muslims from being born.’ (Female, pt. 40, 47 years old)

‘I had no option than to marry them, I had no choice’. As she recounted her horrific experiences.

‘[*I*] spent 11 months in one of the BH camps inside Sambisa Forest. Many women were forced to marry and sexually assaulted by the captors under brutal, inhumane situations. Lots of us were in severe physical pain, sexually transmitted diseases, pregnancies, and desolate conditions … women who declined to marry fighters were humiliated and beaten while others were compelled and deployed with improvised explosive device [IEDs] on a suicide mission against their wish as punishment for their action. A lot of girls are kept as sex slaves, and many [*took their lives or*] became pregnant for their rapist.’ (Female, pt. 41, 24 years old)

#### Sub-theme 4: Perpetrators, reign of terror among the non-Muslim women

On the testimonies of survivors who escaped from BH captivity, the majority of the *rijale* (caliphate fighters) they encountered during their ordeal, who abducted, converted, transferred, imprisoned, maltreated, tortured and abused them, were mostly Kanuri-Muslims; indigenous Muslims collaborators and few foreign fighters and some were Hausa-Fulani faithfuls.

‘Most of the insurgents were between the age of 17–30, and few were considerably in their 50s. Some of the fighters were captured, converted and compelled to join the group. Lots of them could be considered novice who do not even know the basic tenets of the religion they are defending.’ (Female, pt. 08, 27 years old)

A former BH convert revealed that,

‘Enslaving the families of the “kaffir” and taking their girls as well as their women as concubines is a firmly established part of the Shariah … non-Muslim women who were captured and brought into the Dar al-Islam [*abode of Islam*] are permissible to the faithful’s … If she has not known a man, we can have intercourse with her without further delay after owning her. It’s also permissible to presents female captives and slaves as gifts, for they are nothing but mere chattels [*properties*] that can be bought and sell at will … also permissible to have intercourse with female slave who has not reached puberty stage, if she is fit for intercourse; but if she is not clearly fit for sexual relationships, then it is alright to enjoy her without sex.’ (Male pt. 43, 38 years old)

### Theme 2: Rejection, stigma and trauma among non-Muslim survivors

The stigma associated with rape within the non-Muslim communities and the fear of being rejected by families and communities for women and girls who disclose sexual abuses could empirically spell out the small number of first-hand responses.

‘… Even acknowledging abduction by BH could put girl’s marital future in jeopardy. Lack of care and support for displaced non-Muslims who have experienced sexual abuses … including trauma have discouraged women and girls from reporting sexual violence suffered in captivity … consequently [*led to mental injury*] and deaths.’ (Female, pt. 01, 24 years old)

‘When we were brought to the camp at Maiduguri, we received all sorts of insults, some even called us BH wives. They even accused us of being spies. One elderly woman told me that, she is always scared whenever new people arrived to the camp, because what follow next is bomb blast … They thought we had control over what we have been through in captivity. How can a woman say such things to fellow women? They have no idea of the magnitude of violation and traumatic tortures we had survived or went through. We were turned into an object of mockery and topics for discussions. people in the camp were not helping us at all. They made our lives even worst. We felt bad, without peace of mind in the camp. Later, we were taken out of the camp to another, that was being under the control of Christian Association of Nigeria [*CAN Centre*] … people may be afraid to say, but even in camps, in Maiduguri, we were treated differently based on ethnicity and religious identity. Because the moment we enter the camp, all of us started singing worships songs to the glory of Almighty … I believed it was because of our worships song they transferred us to CAN Centre. There, we were not abused, they only showed us love.’ (Female, pt. 07, 26 years old)

‘BH has ruined our lives. My mother and I were raped, and my mother was said to have given birth while being held by the BH; I don’t know her whereabouts my younger sister and the baby. What will be happening to them in captivity. I can’t say, if I may ever see them again in this wicked world.’ (Female, FGDs)

‘… Women are the ones that suffers more. Often described and stigmatised as BH wives. The most traumatic is watching some of my fellow survivors rejected by their bloods and loved ones, who supposed to comfort them and give solace. Meeting my family at Jos, reminded me of the old feelings as they received me with open arms. A lot of people came from different churches and prayed for me. That changes my thought very much. This was what you cannot get in camps at Maiduguri. Such remained me of love and my life at Kwada before BH attack. We played with friends and go with my parents to the farms.’ (Female, pt. 10, 29 years old)

‘I lived in constant fear and experience lots of nightmares, like being dragged away with many non-Muslim girls. I am troubled by an anxiety and fear whenever I recalled my times in BH captivity. The panic, feeling and shortness of breath that go with these memories worsen when I share my traumatic experiences with other survivors from my community, who are also scattered, homeless, since 2014 … Sometimes I wonder how a whole community will be able to survive and living like this on exile, reflected emotionally. All I hope for, and belief is that 1 day I will be able to give evidence to the authorities about what I and others like me experienced. Sometimes I think as if my life is ended.’ (Female, pt. 13, 26 years old)

‘… I always recall how my village was burnt down, the slaughtering of men, and putting their heads on the chest, and the nightmares of hundreds innocent women and girls who have been abducted by those devils, could not get out of my head.’ (Female, pt. 20, 18 years old)

Survivors who were subjected to sexual abuse while in BH captivity show signs of trauma in camp. One survivor said during focus group discussion:

‘[*I*] can’t sleep at night because I remember how BH were beheading those that refused conversion to Islam, and how they were raping me and other girls. I want to forget about it but I can’t, the memories always come. I want to travel far until things get better; I don’t want to be abducted again.’ (Female, FGDs)

‘Even those who were not captured during the onslaught of BH, are still suffering from the carnage … I still cannot believe that I am alive … when I first came back from BH captivity … my family said, I often wake up the family in the middle of the night screaming … I was forced to watch as BH fighters killed lots of people when they took over Chibok and was threatened when I cried over the deaths. The violation we went through in captivity is something I do not want to remember or share, because of the stigma and shame, but thank God for my family.’ (Female, pt. 26, 30 years old)

‘I am haunted by the memories of tortures, beatings, rape and frequent killings of non-Muslims, who refused to join the insurgent group. It was barely a year after, that we were rescued from BH captivity. The Islamic group still abducted over 100 schoolgirls from the town of Dapchi, in Yobe state. A month later all were freed except one, (Leah Sharibu), the only Christian girl among the abductees, who reportedly refused to convert to Islam. That news of Leah, who was denied freedom because of her Christian faith brings back terrible memories, of what we went through after our abduction in 2014, at school in Chibok. Is a fact that, BH wants to convert or exterminate non-Muslims from the region, but Jehovah, would not make that happen.’ (Female, pt. 30, 23 years old)

‘I could not sleep for a week when I heard about what happen to Leah, because I didn’t want her to go through what we went through in the hands of those wicket souls. I always panic and get scared whenever I hear Muslims calls to prayer, or gunshots, even from a far.’ (Female, pt. 31, 24 years old)

The following sub-theme was created from Theme 2.

#### Sub-theme 1: On the road to recovery

The intense trauma of what non-Muslims have been through is another reason why recovery will take a long time:

‘Participants collectively revealed that insurgents, have implanted so much fear and distrust in the hearts of non-Muslims that they no longer even trust own brother Muslim …how can you easily forget someone that led an enemy to you? Killed the husband, burnt down the houses, carted away food stuff and valuables, and abducted your women, girls and children for religious reason.’ (Male, FGDs)

Another participant mentioned during focus group that:

‘[*M*]y children were so afraid whenever they hear Muslims call to prayers at Jos, they slept with their hands over their ears. Even now, some of my children still run to me whenever they hear bang. But with time they began to adapt and regained their life back.’ (Male, FGD, Maiduguri)

‘… I’m now free, but everything else [*I had or knew as family*] before has gone … I hate to see every man, and could not sleep both day and night. Whenever, I saw men in a group, I got panic and scared … I also met many other women and girls who went through similar ordeal. The experiences we shared together made me stronger. Though, some females under treatment did not want to marry, or even consider relationships with men again. I decided to be strong in the Lord and I even made new friends, in the camp … took part in a trauma healing programme run by Christian Persecution Charity ‘Open Doors’. After the program, I got peace for myself as a lone survivor. I appreciated God for that encouragement they gave to me at that time.’ (Female, pt. 07, 26 years old)

‘My father younger brother and community members struggled to accept me when I arrived IDPs camp in Maiduguri…Also, people in the camp called me BH wife. People avoided me; they did not sit with me nor eat with me in the camp … they were frightened by my existence. I really felt bad when I was labelled dangerous, and I had no one beside me, no one to talk or share my feeling with. I felt disappointed, I could not understand why people I hoped would take me in and support me totally disassociated themselves from me. I Felt isolated and alone, from being rejected and stigmatised by my own relative and other community members. I even thought of returning to the insurgents, perhaps they will accept me back … The International non-governmental organisation invited some people that includes my relative to participate in project and become a community peacebuilder, carry out to reduce stigmatisation against women and girls … After the second day of training session, my father’s brother deeply regretted his actions and reconciled with me … training session obviously served as an eye opener for him and others, who rejected their own for something she had no control over …’ (Female, pt. 13, 26 years old)

However, regardless of clearly articulated humanitarian needs and psychosocial counselling to support survivors of gender-based violence, primarily women, girls and children formerly abducted, is lacking. The child protection unit within the non-Muslim communities of southern Borno has seen nothing tangible, making it hard to fully support the humanitarian needs and psychosocial counselling for the dislodged communities on their long road to recovery.

## Discussion

The BH has been known for consistently capturing, raping and sexually violating women and girls whom they considered to be disposable property. The experiences of the participants from the findings show that the massive atrocities committed by BH in its array of brutality included (but not limited to) physical torture, forced displacement, gang rape and forced marriages as well as forced conversion to Islam. Such practices against non-Muslim women and girls cause serious psychological devastation or mental harm. Such harm and mental grief was aggravated by torturous tactics such as systematically separating wives from their husbands, children from their mothers, forcefully taking their daughters into sexual slavery, male children forcefully indoctrinated as well as being forced either to witness their executions or to watch them being mistreated and taken to unknown destinations.

The ensuing trauma was even exacerbated by actions such as mothers being raped in front of their daughters, and vice versa. In the long term, the bodily and sexual violence, along with the serious mental wounds which non-Muslim women and girls over the age of seven endured at the hands of BH, inflicted severe bodily and mental suffering on the survivors. The consequences of physical and mental wounds that BH inflicted on captured non-Muslim women and girls surpassed the act of rape itself.

The implications of this trauma have been reviewed from the experiences of other groups that have been victims of genocide, such as in Rwanda and former Yugoslavia.

## Genocide against the non-Muslim

Under the Rome Statute, ‘genocide’ means any of the following acts committed with the intent to destroy, in whole or in part, a national, ethnical, racial or religious group, as such: (1) killing members of the group; (2) causing serious bodily or mental harm to members of the group; (3) deliberately inflicting on the group conditions of life calculated to bring about its physical destruction in whole or in part; (4) imposing measures intended to prevent births within the group; or (5) forcibly transferring children of the group to another group.^16^

Boko Haram abuse highlighted in the findings show that their treatment of Muslims was different to their treatment of what they call ‘kirdi’ (non-Muslim). In BH eyes, and in their own words, non-Muslim women and girls are ‘Merely chattels (properties), that could be bought and be disposed of’ disclosed participants. According to Paul Robinson, the Chief Executive Officer (CEO) of Release International, tens of thousands of non-Muslims are being attacked and driven away from their inhabitants by the ongoing persecution, as the global death toll is rising rapidly, and the world is watching.^17^

In 2016, the new factional leader of the sect (ISWAP), Abu Musab Al-Barnawi, had ordered the group’s fighters to attack Christians by ‘booby-trapping and blowing up every church that we can reach, and killing all of those who we find from the citizens of the cross’ while ending attacks on mosques and markets used by ordinary Muslims.^18^

### The killing of the non-Muslims

The BH group has been killing non-Muslim men and women; in addition to the chapeau element of intent to destroy, genocide can be perpetrated by killing one or more persons belonging to a group.^16^

As captured in the findings, ‘by virtue of your identity as non-Muslims and unwillingness to convert to Islam, a lot of girls were abused and tortured to death in different places’.

Johnnie Moore, co-author of ‘The Next Jihad’, declared that non-Muslim communities have been ravaged by jihadist extremists in parts of northern Nigeria and that thousands of non-Muslim worship centres have been destroyed, children killed, clergies beheaded, villages and fields set on fire by the tens of thousands, with individuals being targeted for their Christian faith alone.^19^

Former U.S. Congressman Frank Wolf called the campaign against Christians in northern Nigeria a ‘deliberate genocide’. Nigeria was becoming the ‘biggest killing ground of Christians in the world’.^20^

Boko Haram has intentionally killed hundreds of non-Muslims as part of its campaign to rid the north of infidels. As reflected in the literature, in a report titled: Nigeria is becoming world’s ‘biggest killing ground of Christians’, the International Christian Concern (ICC) revealed that:

[*B*]etween 50 000 and 70 000 non-Muslims have been murdered in cold blood, in the last decade in Nigeria, the most populous black nation on the continent by the BH and other jihadists.^21^

In 2014, BH ‘rijale’ summarily killed hundreds of non-Muslim men and teenage boys when the victims refused to convert to Islam. Free will inhumanity was exhibited in the awful mass killing of the non-Muslim community of Chibok by BH terrorists. Participants said that mass executions occurred in Bwarakila, Kuburmbula, Kwada, Gatamarwa, Kagilmari, Kwatiya, Kautikari, Kuburivu, Bulakar villages. They also set some men on fire at Bolakeli, heating, flogging and telling them to profess Islam or die; but the men refused to convert and burnt in the fire.

In October 2014, as captured in the findings, ‘BH fighters massacred an undefined number of non-Muslim women and children in Bwaftari and stoned others to death after putting them in a hole’. Reports of these killings are based on participants’ experiences. None of the non-Muslim male victims who were captured and led away, since 2014 have been found. Large mass graves have been found in Madagali since 2014, where the researcher suspects that over 4000 non-Muslims and apostate were buried. Although many of the non-Muslim communities are still deserted, many more could be discovered after the war.

According to Paul Robinson, CEO of Release International:

‘[*B*]oko Haram and ISWAP have both pledged to kill non-Muslims (Christians), and heavily armed Fulani militants are driving non-Muslims villagers from the north. Release International’s partners warn of genocide in the making.’^16^

In addition, Release Partner Hassan John faulted the Nigerian government for failing to halt the violence against non-Muslims. He said, ‘the government, by design or omission, is turning a blind eye to the carnage’.^22^

Several non-Muslim women and girls, held captive by BH in their ‘*daular*’, killed themselves before being forcibly married to BH fighters. Some drank poison to avoid marrying BH insurgents in Sambisa Forest, where Kibaku women and girls were gathered, tortured and forcefully married off. International Criminal Tribunal for the former Yugoslavia (ICTY) jurisprudence holds that the suicide of a person may amount to killing where the accused’s acts or omissions induced the victim to take actions that resulted in their death and that their suicide was either intended or was an act of a type which a reasonable person could have foreseen as a consequence.^23^

Boko Haram has carried out the prohibited killing of members of a protected ethnic and religious group (Kibaku communities) of southern Borno.

## Boko Haram has caused serious bodily and mental harm to non-Muslim women and girl captives

Acts leading to serious bodily or mental harm can comprise, but is not necessarily confined to, acts of rape, torture, sexual violence, inhuman or degrading treatment.^16^ Besides the chapeau element of intent to destroy, causing serious bodily or mental harm to one or more persons belonging to a particular group constitutes genocide.^24^

### Rape and sexual violence

After the Akayesu decision in the International Criminal Tribunal for Rwanda (ICTR), rape and sexual violence can be interpreted as serious physical and psychological harm, if committed with specific plans to destroy a protected group, in whole or in part.^25^ The court in Akayesu declared that rape and sexual violence can constitute a form of genocide, indeed by inflicting serious bodily and mental harm on the victims. The International Court of Justice has also recognised that rape and other crimes of sexual assault can amount to genocide, that is, by causing serious physical harm.^26^

Boko Haram fighters systematically rape and abuse non-Muslim women and girls as young as 10 years. Moreover, there is hard evidence of such rapes occurring from participants, who display both bodily and psychological suffering. The consequences of physical and mental wounds that BH committed against captured non-Muslim female victims surpassed rape itself. From the views of the survivors and perpetrators, captured non-Muslim women and girls are put through unabated sexual violence in a climate of impunity and judicial dysfunction.

Besides rape, forced impregnation causes ‘serious psychological wounds’ to members of a group by forcing women and girls to become pregnant and bear children for their assailants.^27^ For instance, non-Muslim survivors who were impregnated through rape while in captivity suffer, psychologically scarred by the pregnancy as the fathers of the children are unknown; they are also unable to build up normal sexual relationships or a family with persons of their choice.

These elements constitute evidence that BH has committed genocide against non-Muslim minority ethnic groups by causing serious physical and mental suffering on non-Muslim women and girls.

### Torture, inhuman and degrading treatment

At the point of capture, non-Muslim women and children experienced mental harm resulting from being separated from their men and being forced to witness their executions or to watch them being mistreated and taken to an unknown destination.

Boko Haram fighters savagely beat abducted non-Muslim women and girls if they resist their advances, try to escape or stop BH insurgents from taking their children out of their care. In addition, serious mental grief is being inflicted on non-Muslim women as a result of insurgents forcefully taking their daughters into sexual slavery and their male children forcefully indoctrinated and enlisted as BH fighters. For several non-Muslim women with no knowledge of where their children are or what situations they are living under, the trauma is all-consuming. The bodily and sexual violence, along with the severe mental wounds, which non-Muslim women and girls over the age of seven have gone through at the hands of BH, reach the level of torture, inflicting severe bodily and mental suffering to the survivors.

Boko Haram also causes severe bodily and psychological wounds to non-Muslim girls and teenage boys. This suffering resulted from the violent separation of non-Muslims from their parents and siblings, forced conversions, ensuing indoctrination, enrolment and brutal training, which comprises battering, beheading, training on suicide bombing, teachings of ‘no God but Allah’ martyr and watching violent videos; subsequent utilisation to fully engage in hostilities; attacking either former host or mother communities. Through these crimes and abuse, BH plans to totally rid the north of perceived infidels and destroy the children’s identity as non-Muslims. Acts of this nature constitute war crimes.

## Limitations

A sociological study in such a deserted and volatile environment is assumed to face several ethical issues and practical challenges. The researcher, for that reason, wishes to admit that some ethical and functional difficulties in line with field research in a fragile and conflict environment were experienced during data collection. For example, some of the survivors initially expected that participation in the study would attract financial gains. Therefore, some people who were not eligible made efforts to influence the selection process.

There was also a lack of archival documents. This limitation emerging from the lack of prior studies limits the contrast and arguments of the study.

The research study employed a qualitative research design; the findings cannot be generalised to all BH survivors as they cover a specific area.

A significant challenge experienced was that the researcher could not collect data in some unsafe communities. For example, during data collection, just 500 m from the study location BH attacked and abducted some women while fetching firewood. Consequently, they killed a vigilante on a rescue operation.

Finally, it was not easy to access the high-profile non-Muslim survivors like clergy or senior government officials who attracted media attention while in BH captivity.

### Recommendations

The international community needs to acknowledge that BH has committed the crimes of genocide, against the non-Muslims ethnic minorities in southern Borno and non-Muslims in north-eastern Nigeria in general; and to also strengthen the human rights capacity in Nigeria to respond more effectively to allegations of human rights abuses and violations.

The government, as a matter of urgency, should establish a victim-centred scheme to address the right of survivors to religious freedom, justice and reparations as a necessity. The fundamental political and social conditions that permit BH to exploit religious and ethnic break-up in north-eastern Nigeria should be challenged. Not doing so risks causing a repetition. Government should provide remedies for women and girls whose human rights have been violated, and have experienced sexual violence. These women and girls require full access to psychosocial counselling, rehabilitation and social reintegration, provisions of sexual and reproductive health services, including HIV therapy. The government should take necessary measures to safeguard the life and health of women and girls, and to address stigma against women and girl survivors of sexual violence and their children, through orientations and seminars, speaking out against negative statements and, making sure that communications do not reinforce stereotypes.

## Conclusion

Evidence documented in this article has shown that BH has committed, and continues to commit, the crime of genocide and war crimes against the non-Muslim communities of southern Borno. The Islamic insurgents tried to destroy the ethnic minorities ‘physically and mentally’ and threatened many different communities with the final offer to ‘convert or be killed’.

Therefore, the reality is the same. The non-Muslim communities of southern Borno had experienced some of the worst unreported mass atrocities known to humankind. They desperately need psycho-social support and the help of the international community who should intensify efforts to ensure justice for the survivors and those responsible, directly or as sponsors, commanders or collaborators are brought to justice.
